# Impact of factor V Leiden on outcomes following coronary artery bypass grafting: a national inpatient sample analysis

**DOI:** 10.3389/fcvm.2025.1678264

**Published:** 2025-12-05

**Authors:** Alec Czaplicki, Ashley Rensted, Shannon Blee, Eli Blaney, Saif Zurob, Abubakar Tauseef, Ali Bin Abdul Jabbar

**Affiliations:** 1 Creighton University School of Medicine, Omaha, NE, United States; 2 Department of Medicine, Creighton University School of Medicine, Omaha, NE, United States

**Keywords:** coronary artery bypass grafting, factor V leiden, mortality, coronary artery disease, length of stay

## Abstract

**Background:**

Coronary artery bypass grafting (CABG) is commonly used for revascularization in patients with advanced coronary artery disease. Factor V Leiden (FVL) is a hereditary hypercoagulable state in which factor V is resistant to activation by activated protein C, causing activation of prothrombin and an increased propensity for thrombotic events. In this study, we aim to establish whether FVL increases in-hospital mortality following a CABG procedure.

**Methods:**

The National Inpatient Sample was used to extract data on patients who underwent a CABG procedure between 2017 and 2022. Patients were grouped based on a diagnosis of FVL (*N* = 2,095) or not (*N* = 1,142,735). Propensity scores were estimated using logistic regressions and stratified into six subclassifications, and generalized linear models were used to assess differences in in-hospital mortality, length of stay, and total cost.

**Results:**

A higher proportion of patients with FVL were female (*p* = 0.006) and white (*p* < 0.001). There was no significant difference in in-hospital mortality between those with and without FVL who underwent a CABG procedure (95% CI 0.52–1.78, *p* = 0.89). Those with FVL had 7% longer hospital stays (95% CI: 1.0–1.13, *p* = 0.025) but did not incur higher costs for their stay (95% CI: 0.92–1.08, *p* = 0.97) compared to those without FVL.

**Conclusions:**

FVL is not associated with increased risk of in-hospital mortality following a CABG procedure. This finding can help guide providers considering CABG for revascularization in patients with FVL.

## Introduction

Coronary artery bypass grafting (CABG) is a procedure done to restore adequate blood flow to the myocardium by harvesting vasculature from elsewhere in the body and using it to bypass atheromatous coronary arteries. Almost 400,000 CABG procedures are performed in the United States each year (1). Because other methods for revascularization, like percutaneous coronary intervention (PCI), exist, CABG procedures are reserved for coronary artery disease (CAD) of high severity or complexity. The decision to perform a CABG is complex and dependent on many factors, often requiring a multidisciplinary team of providers. The American College of Cardiology Foundation/American Heart Association Task Force recommends CABG for revascularization in patients with ≥50% left main stenosis, three-vessel disease with ≥70% stenosis, two-vessel disease involving the left anterior descending coronary artery, ≥70% stenosis with refractory angina despite maximal medical therapy, or ≥70% stenosis with ischemia-related ventricular tachycardia after sudden cardiac death (2). A 2020 study of patients on Medicare who underwent CABG procedures showed that the most common postoperative complications were new-onset atrial fibrillation (30.0%), prolonged ventilation (12.3%), renal failure (4.5%), reoperation (3.5%), and cerebrovascular accidents (1.9%), with one of these complications occurring in 41% of patients (3).

Factor V Leiden (FVL) is a hereditary thrombophilia caused by a point mutation in which the factor V cleavage site is eliminated, thus preventing activated protein C from binding and inactivating factor V (4). This results in further activation of the coagulation cascade and an increased propensity for thromboses in affected individuals. FVL is inherited in an autosomal dominant fashion, and those with heterozygosity of the mutation have a 7-fold increase in lifetime thrombosis risk compared to the general population (4). FVL is seen equally in males and females and is the most common hereditary thrombophilia, affecting between 3% and 8% of Caucasian Americans and Europeans (5).

Thrombotic events, including graft thrombosis, deep vein thrombosis (DVT), and cerebrovascular accidents, are all feared complications of CABG procedures. There is a gap in the current literature regarding the risk of cardiac procedures, such as CABG, in patients with FVL, given their increased propensity for thromboses. A previous study of patients heterozygous for the factor V Leiden mutation showed a higher rate of early saphenous vein graft occlusion after CABG compared to non-carriers (5/11 carriers vs. 18/89 non-carriers), suggesting a possible link between factor V Leiden and postoperative arterial thrombosis (Moor 1998) (6). Additionally, a 2009 study investigating 14 patients undergoing cardiac surgery, which consisted of 10 CABG procedures, found that symptomatic patients with FVL had increased thromboembolic events (7); however, the small sample size and single-center nature are significant limitations of these studies. A 2023 systematic review of patients undergoing noncardiac surgery showed that patients with FVL have an increased risk for thromboembolic events in both the perioperative and postoperative periods when compared to patients without FVL (7, 8). In contrast, other studies have shown that FVL is not associated with an increased risk for atherothrombotic events in patients with treated coronary heart disease. These studies found that after controlling for age, FVL has no correlation with an increased risk for acute myocardial infarction (9, 10). Moreover, larger cohort studies have not consistently demonstrated a statistically significant increase in arterial thrombotic events or mortality in factor V Leiden carriers after CABG procedures.

Given the paucity of literature exploring the impact of FVL on outcomes following CABG procedures, we aimed to conduct a large study that investigated whether FVL affects in-hospital mortality, length of stay, and total costs following a CABG procedure. We hypothesized that given FVL's increased propensity for thrombotic events, those with FVL would have higher rates of in-hospital mortality and complications due to thrombogenicity, leading to longer hospital stays and increased total costs.

## Materials and methods

### Data source and ethics statement

Data from the National Inpatient Sample (NIS) were abstracted from the years 2017–2022. This database is one of the largest publicly available inpatient healthcare databases in the United States and represents approximately 35 million hospitalizations nationally (11). It was developed as part of the Healthcare Cost and Utilization Project (HCUP).

### Patients

All included hospitalizations were from patients ≥18 years old who received a CABG procedure. Specifically, patients who underwent CABG with the International Classification of Diseases-10 Procedure (ICD-10-PMS) codes 0210, 0211, 0212, and 0213 were included; these include single, double, triple, and four or more artery bypasses, respectively. Hospitalizations for FVL and non-FVL were differentiated based on the primary discharge diagnosis. Similar to prior NIS studies, three racial and ethnic groups with small sample sizes (Asian or Pacific Islander, Native American, and Other) were combined into a single “other” group for ease of analysis (12). The other three HCUP race/ethnicity groups (White, Black, Hispanic) were left unchanged for the study. “White” group refers to non-Hispanic White patients, “Black” group refers to non-Hispanic Black patients, and “Hispanic” group refers to Hispanic patients of all races and origins.

### Exposure

The FVL-group hospitalizations were identified using International Classification of Diseases-10 Clinical Modification (ICD-10-CM) code D68.51.

### Outcomes

The primary study outcome was in-hospital mortality. Secondary outcomes included hospital length of stay (LOS) and total hospital cost. Hospital cost was inflation-adjusted to mid-year 2024 US dollars (13).

### Statistical analysis

Continuous variables are presented as mean and standard error, compared using Kruskal–Wallis test, whereas categorical variables are presented as percent, compared using the Pearson's Chi Squared with Rao-Scott adjustment. Differences in all-cause in-hospital mortality were evaluated using generalized linear models using a quasi-binomial distribution. Hospital length of stay and inflation-adjusted total hospital cost were evaluated using generalized linear models using a quasi-Gamma distribution with a log link function to account for positive skew and zero-values.

To minimize selection bias, we used propensity score stratification. Propensity scores were estimated by a logistic regression model using FVL as the outcome and matching for age, biological sex, race, insurance status, hospital teaching status, income quartile by zip code, weekend/weekday, elective admission, hospital bed size, hospital region, Elixhauser sum (number of Elixhauser comorbidities present), chronic obstructive pulmonary disease, cardiac arrhythmia, valvular disease, pulmonary circulation disorders, PVD, hypertension, paralysis, other neurological conditions, uncomplicated and complicated diabetes, hypothyroidism, renal failure, liver disease, peptic ulcer disease, acquired immunodeficiency syndrome, lymphoma, metastasis, solid tumors, rheumatoid arthritis, obesity, weight loss, fluid/electrolyte disorders, blood loss anemias, deficiency anemias, alcohol use disorder, 1-vessel CABG, 2-vessel CABG, 3-vessel CABG, 4-vessel CABG, previous pulmonary embolism (PE), previous cardiovascular disease (CVD), previous DVT, and smoking status. The propensity score was subclassified into six groups using the MatchIt package in R (14, 15). The quality of the match was evaluated by comparing standardized mean differences and variance ratios for the covariates used to estimate the propensity score. The decision to retain nonlinear forms was determined using likelihood ratio test (16). All analyses were conducted using R version 4.4.2, accounted for the NIS sampling design, and were weighted to estimate national-level effects. Two-tailed *p* < 0.05 was defined to indicate statistical significance.

## Results

### Baseline characteristics

Data was pulled from the hospital stays of 1,144,830 patients who underwent CABG procedures. Of these patients, 2,095 had a history of FVL (FVL group) and the remaining 1,142,735 did not (non-FVL group). The average ages were 66.0 ± 0.5 and 66.3 ± 0.0 for the FVL and non-FVL groups, respectively (*p* = 0.697). The FVL group was 29.8% female and 70.2% male, whereas the non-FVL group was 24.1% female and 75.9% male (*p* = 0.006). A higher percentage (91.9%) of patients in the FVL group were White compared to the non-FVL group (75.5%). The non-FVL group had a higher percentage of other races than the FVL group, including Black patients (6.8% vs. 1.4%), Hispanic patients (7.6% vs. 1.2%), Asian or Pacific Islander (0.5% vs. 3.5%), and Native American (0.5% vs. 0.6%) (*p* < 0.001)*.* A slightly higher percentage of patients in the FVL group had insurance coverage through Medicare (58.7% vs. 56.2%) and private insurance (32.9% vs. 30.0%) than the non-FVL group, although the statistical significance is unknown. However, a high percentage of the non-FVL group had coverage through Medicaid than the FVL group (7.5% vs. 1.0%). A similar percentage of patients in each group received their care at urban teaching hospitals [88.1% (FVL) vs. 84.2% (non-FVL)], urban non-teaching hospitals [10.0% (FVL) vs. 13.1% (non-FVL)], and rural hospitals (1.0% (FVL) vs. 2.7% (non-FVL) (*p* = 0.088). Finally, there were significant differences in the geographical regions of the two patient groups. Specifically, the FVL group had higher percentages of patients from the Northeast [19.3% (FVL) vs. 16.6% (non-FVL)] and Midwest or North Central regions [33.4% (FVL) vs. 23.2% (non-FVL)]. The non-FVL group had a higher percentage of patients from the South [31.2% (FVL) vs. 43.4% (non-FVL)], and West regions [15.5% (FVL) vs. 16.8% (non-FVL); *p* < 0.001] All results described above are presented in Table 1.

**Table 1:** Baseline demographic characteristics.

Category	Variable	Overall (*N* = 1,144,830)	FVL (*N* = 2,095)	Non-FVL (*N* = 1,142,735)	*p*-value
	Age	66.3 ± 0.0	66.0 ± 0.5	66.3 ± 0.0	0.697
Biological Sex	Female	24.1%	29.8%	24.1%	0.006
	Male	75.9%	70.2%	75.9%	
Race	White	75.5%	91.9%	75.5%	<0.001
	Black	6.8%	1.4%	6.8%	
	Hispanic	7.6%	1.2%	7.6%	
	Asian or Pacific Islander	3.3%	0.5%	3.3%	
	Native American	0.6%	0.5%	0.6%	
	Other	1.0%	0.2%	1.0%	
Insurance Status	Medicare	56.2%	58.7%	56.2%	0.018
	Medicaid	7.5%	1.0%	7.5%	
	Private Insurance	30.0%	32.9%	30.0%	
Hospital Teaching Status	Urban Teaching	84.2%	88.1%	84.2%	0.088
	Rural	2.7%	1.0%	2.7%	
	Urban Nonteaching	13.1%	10.0%	13.1%	
Hospital Region	Northeast	16.6%	19.3%	16.6%	<0.001
	Midwest or North Central	23.2%	33.4%	23.2%	
	South	43.4%	31.2%	43.4%	
	West	16.8%	15.5%	16.8%	

FVL, Factor V Leiden.

Design-based Kruskal–Wallis test and Pearson's *χ*^2^: Rao & Scott adjustment, were the statistical tests used to analyze this data.

The FVL group had a higher proportion of patients with a previous history of DVT (36.0% vs. 2.0%; *p* < 0.001), PE (23.0% vs. 1.2%; *p* < 0.001), cardiovascular disease (14.0% vs. 8.8%; *p* < 0.001), and peripheral vascular disease (20.0% vs. 15.0%; *p* < 0.001) compared to the non-FVL group. Additionally, the mean Elixhauser comorbidity index was higher in the FVL group (5.4) compared to the non-FVL group (4.7; *p* < 0.001). Finally, a lower proportion of the FVL group (39.0%) had a history of smoking than the non-FVL group (48.0%; *p* < 0.001) All results described in this paragraph are presented in Table 2.

**Table 2:** Baseline clinical variables.

Variable	Overall (*N* = 1,144,830^a^)	FVL (*N* = 2,095^a^)	Non-FVL (*N* = 1,142,735^a^)	*p*-value^b^
Previous PE	14,050 (1.2%)	485 (23%)	13,565 (1.2%)	<0.001
Previous CVD	101,100 (8.8%)	295 (14%)	100,805 (8.8%)	<0.001
Previous DVT	23,975 (2.1%)	755 (36%)	23,220 (2.0%)	<0.001
Elixhauser sum	4.68 ± 0.01	5.43 ± 0.10	4.68 ± 0.01	<0.001
Chronic pulmonary disease	247,565 (22%)	490 (23%)	247,075 (22%)	0.387
Cardiac arrhythmia	580,260 (51%)	1,070 (51%)	579,190 (51%)	0.874
Valvular disease	293,135 (26%)	610 (29%)	292,525 (26%)	0.096
Pulmonary circulation disease	70,210 (6.1%)	170 (8.1%)	70,040 (6.1%)	0.092
Peripheral vascular disease	172,975 (15%)	410 (20%)	172,565 (15%)	0.011
Uncomplicated hypertension	534,690 (47%)	925 (44%)	533,765 (47%)	0.297
Uncomplicated diabetes	171,435 (15%)	215 (10%)	171,220 (15%)	0.007
Complicated diabetes	393,885 (34%)	640 (31%)	393,245 (34%)	0.094
Hypothyroidism	130,820 (11%)	245 (12%)	130,575 (11%)	0.864
Renal failure and disease	253,995 (22%)	505 (24%)	253,490 (22%)	0.342
Liver disease	50,955 (4.5%)	100 (4.8%)	50,855 (4.5%)	0.748
Metastatic cancer	2,845 (0.2%)	15 (0.7%)	2,830 (0.2%)	0.054
Solid tumor without metastasis	16,100 (1.4%)	40 (1.9%)	16,060 (1.4%)	0.380
Obesity	345,310 (30%)	685 (33%)	344,625 (30%)	0.253
Complicated hypertension	491,475 (43%)	825 (39%)	490,650 (43%)	0.150
Atrial Fibrillation	412,135 (36%)	770 (37%)	411,365 (36%)	0.749
Dyslipidemia	928,715 (81%)	1,620 (77%)	927,095 (81%)	0.053
Smoking	554,955 (48%)	825 (39%)	554,130 (48%)	<0.001
Previous MI	201,250 (18%)	360 (17%)	200,890 (18%)	0.829

FVL, Factor V Leiden; PE, pulmonary embolism; CVD, cardiovascular disease; DVT, deep vein thrombosis; MI, myocardial infarction.

Design-based Kruskal–Wallis test and Pearson’s χ^2^, Rao & Scott adjustment, were the statistical tests used to analyze this data.

a *n* (%); Mean ± SE.

b Pearson's χ² with Rao & Scott adjustment; Design-based Kruskal–Wallis test.

### Propensity score stratification balancing

Propensity scores were calculated based on a scheme of six subclassifications. 40 observations were dropped from the FVL group due to incomplete cases. Similarly, 29,799 observations were dropped from the Non-FVL group due to incomplete cases. All remaining cases were matched, keeping 396 observations in the FVL group and 205,866 observations in the Non-FVL group. The effective sample size was 396 observations in the FVL group and 15,424 observations in the Non-FVL group.

The variance ratio of age decreased from 1.0675 to 1.0134. The standard mean difference (SMD) of female patient admissions reduced from 0.1064 to −0.042. For race, the SMD of White patients reduced from 0.9523 to 0.0985; Black, −0.6963 to −0.0995; Hispanic, −0.6599 to −0.0656; Asian or Pacific Islander, −0.4170 to −0.0294; Native American −0.0005–0.0011; and Other, −0.1436–0.0065. For insurance, the SMD of Medicare admissions increased from 0.0289 to 0.0312; Medicaid, −0.1587 to −0.0028; Private insurance, 0.0397 to −0.0308. The SMD of admissions with history of PE reduced from 0.5256 to 0.0792. However, there remained residual confounding for admissions with a history of PE (*p* = 0.032). For previous CVD, the SMD reduced from 0.1188 to −0.0036. The SMD of previous DVT thrombosis decreased from 0.7222 to 0.0249. The variance ratio of the Elixhauser sum increased from 0.9957 to 1.1166. Additionally, residual confounding remained for admissions with documented history of drug abuse (*p* = 0.001), depression (*p* < 0.001), and complicated hypertension (*p* < 0.001). The propensity balancing results are presented in Table 3.

**Table 3:** Means of FVL and Non-FVL groups before and after propensity score stratification.

Variable	Pre-PSS			Post-PSS		
	Means FVL	Means Non-FVL	Std. Mean Diff.	Means FVL	Means Non-FVL	Std. Mean Diff.
Age^a^	66.0712	66.7104	−0.063	66.0712	65.5311	0.0532
Female	0.2955	0.2470	0.1064	0.2955	0.3147	−0.042
Male	0.7045	0.7530	−0.1064	0.7045	0.6853	0.0420
Race—						
White	0.9683	0.8016	0.9523	0.9683	0.9511	0.0985
Race—						
Black	0.0079	0.0696	−0.6963	0.0079	0.0167	−0.0995
Race—						
Hispanic	0.0106	0.0780	−0.6599	0.0106	0.0173	−0.0656
Race—						
Asian or Pacific Islander	0.0053	0.0355	−0.4170	0.0053	0.0074	−0.0294
Race—						
Native American	0.0053	0.0053	−0.0005	0.0053	0.0052	0.0011
Race—						
Other	0.0026	0.01	−0.1436	0.0026	0.0023	0.0065
Insured—						
Medicare	0.6174	0.6034	0.0289	0.6174	0.6022	0.0312
Insured—						
Medicaid	0.0449	0.0777	−0.1587	0.0449	0.0454	−0.0028
Insured—						
Private insurance	0.3377	0.3189	0.0397	0.3377	0.3523	−0.0308
Hospital—						
Urban teaching	0.8813	0.8440	0.1152	0.8813	0.8800	0.004
Hospital—						
Rural	0.0158	0.0265	−0.0859	0.0158	0.0178	−0.0156
Hospital—						
Urban non-teaching	0.1029	0.1295	−0.0874	0.1029	0.1023	0.0021
Income—						
25th percentile	0.1926	0.2723	−0.2019	0.1926	0.1964	−0.0097
Income—						
26th to 50th percentile	0.2929	0.2736	0.0423	0.2929	0.2927	0.0003
Income—						
51st to 75th percentile	0.2955	0.2474	0.1053	0.2955	0.2944	0.0024
Income—						
76th to 100th percentile	0.2190	0.2067	0.0299	0.2190	0.2164	0.0062
Non-elective	0.4670	0.4971	−0.0603	0.4670	0.4689	−0.0038
Elective	0.5330	0.5029	0.0603	0.5330	0.5311	0.0038
Region—						
Northeast	0.1926	0.1699	0.0576	0.1926	0.2029	−0.0261
Region—						
Midwest or North central	0.3377	0.2335	0.2204	0.3377	0.3299	0.0167
Region—						
South	0.3087	0.4288	−0.2599	0.3087	0.3076	0.0024
Region—						
West	0.1609	0.1678	−0.0187	0.1609	0.1596	0.0036
Elixhauser sum^a^	5.4327	4.6982	0.3453	5.4327	5.1813	0.1182
Pervious PE	0.2348	0.0120	0.5256	0.2348	0.2013	0.0792
Previous CVD	0.1293	0.0894	0.1188	0.1293	0.1305	−0.0036
Previous DVT	0.3694	0.0209	0.7222	0.3694	0.3574	0.0249

PSS, propensity score stratification; FVL, Factor V Leiden; PE, pulmonary embolism; CVD, cardiovascular disease; DVT, deep vein thrombosis.

a Variance ratios: age, 1.0675 (pre), 1.0134 (post); Elixhauser sum, 0.9957 (pre), 1.1166 (post).

### Mortality

There was no significant difference in in-hospital mortality for patients with FVL after CABG procedures compared to those without FVL (OR: 0.96; 95% CI: 0.52–1.78; *p* = 0.89). Patients who died were more likely to be female (OR male: 0.59; 95% CI: 0.49–0.72; *p* < 0.001) and have history of liver disease (OR: 3.31; 95% CI: 2.48–4.42; *p* < 0.001). Higher Elixhauser comorbidity indexes (OR: 1.75; 95% CI: 1.47–2.10; *p* < 0.001) and age (OR: 1.02; 95% CI: 1.00–1.04; *p* = 0.013) increased the odds of mortality. Admissions where patients died were not more or less likely to have a history of DVT (OR: 0.73; 95% CI: 0.51–1.04; *p* = 0.081) and PE (OR: 0.92; 95% CI: 0.55–1.53; *p* = 0.75). All results described in this paragraph are presented in Table 4.

**Table 4:** In-hospital mortality outcomes.

Variable	Odds Ratio	95% CI	*p*-value
FVL	0.96	0.52, 1.78	0.89
Male Sex	0.59	0.49, 0.72	<0.001
Age	1.02	1.00, 1.04	0.013
Elixhauser Index	1.75	1.47, 2.10	<0.001
Previous CVD	0.80	0.56, 1.14	0.22
Previous DVT	0.73	0.51, 1.04	0.081
Previous PE	*0*.*92*	*0.55, 1.53*	*0*.*75*

FVL, Factor V Leiden; CVD, cardiovascular disease; DVT, deep vein thrombosis; PE, pulmonary embolism.

Generalized linear models were the statistical tests used to analyze this data.

### Length of stay

Admissions lasted on average 7% longer for the FVL group compared to the non-FVL group (95% CI: 1.01–1.13; *p* = 0.025). With every one-point increase in the Elixhauser comorbidity index, patients stayed 9% longer in the hospital (95% CI: 1.08–1.10; *p* < 0.001). Patients undergoing elective CABG procedures stayed 27% fewer days than patients undergoing non-elective procedures (95% CI: 0.72–0.74; *p* < 0.001). All results described in this paragraph are presented in Table 5.

**Table 5:** Length of stay outcomes.

Variable	exp(Beta)	95% CI	*p*-value
FVL	1.07	1.01, 1.13	0.025
Male Sex	0.95	0.94, 0.97	<0.001
Age	1.00	1.00, 1.00	<0.001
Elixhauser Index	1.09	1.08, 1.10	<0.001
Previous CVD	1.00	0.97, 1.02	0.78
Previous DVT	−0.67	−1.0, −0.34	<0.001
Previous PE	0.99	0.96, 1.03	0.70

FVL, Factor V Leiden; CVD, cardiovascular disease; DVT, deep vein thrombosis; PE, pulmonary embolism.

Generalized linear models were the statistical tests used to analyze this data.

### Cost

There was no significant difference in total cost for the hospital admission between the FVL and non-FVL groups (95% CI: 0.92–1.08; *p* = 0.97). With every one-point increase in the Elixhauser comorbidity index, patients had a 13% increase in their total cost (95% CI: 1.11–1.15; *p* *<* 0.001). Additionally, the total cost for hospital admission with elective CABG procedures cost 14% less than admissions with non-elective CABG procedures (95% CI: 0.84–0.87; *p* = 0.28). All results described in this paragraph are presented in Table 6.

**Table 6:** Total cost outcomes.

Variable	exp(Beta)	95% CI	*p*-value
FVL	1.00	0.92, 1.08	0.97
Male Sex	0.98	0.97, 1.00	0.12
Age	1.00	1.00, 1.00	0.029
Elixhauser Index	1.13	1.11, 1.15	<0.001
Previous CVD	0.98	0.95, 1.01	0.25
Previous DVT	0.97	0.94, 0.99	0.012
Previous PE	0.95	0.92, 0.99	0.010

FVL, Factor V Leiden; CVD, cardiovascular disease; DVT, deep vein thrombosis; PE, pulmonary embolism.

Generalized linear models were the statistical tests used to analyze this data.

## Discussion

In this nationally representative sample of in-patient hospitalizations, we did not find evidence of increased in-patient mortality in patients with FVL compared to those without FVL after receiving a CABG procedure. Furthermore, hospital length of stay was longer in patients with FVL compared to those without FVL, but there was no statistically significant difference in the total cost of the hospital stay.

Although it is well-established that FVL increases the risk of thrombosis (4), there was no significant increase in in-hospital mortality among patients with FVL in the current study. A higher proportion of FVL patients in our sample had a history of DVT and PE, which aligns with a previous study that showed DVT and PE as the most common sites of thrombosis in those with FVL (17). However, a history of DVT and PE did not increase the odds of mortality in this study, suggesting that one possible explanation for there being no higher mortality associated with VFL status could be that FVL-associated thromboses do not significantly impact CABG-related mortality. Additionally, a higher proportion of our FVL patients had CVD, yet CVD was associated with lower odds of mortality. This finding is consistent with a 2020 meta-analysis which found no association between FVL and increased atherosclerotic events and mortality in patients with diagnosed and treated coronary artery disease (10).

Another possible explanation for why FVL is not associated with increased post-CABG procedure mortality could be perioperative anticoagulation protocols. Practice guidelines for perioperative management of patients on long-term anticoagulation therapy require careful planning, including possible bridging with low molecular weight heparin or unfractionated heparin (18). Moreover, recent literature suggests that the use of more potent anticoagulants in patients with FVL can reduce the increased risk of venous thromboembolism (VTE) after total hip and shoulder arthroplasty (19, 20). These studies also found no elevated risk of perioperative adverse events, 5-year reoperations, and bleeding events. In addition, one study analyzing patients with FVL who underwent a CABG procedure and received aprotinin, a proteinase inhibitor used in cardiac surgery to reduce bleeding, did not display increased bleeding or postoperative thromboembolic applications even with the combined risk of thrombosis with FVL and aprotinin (21).

A previous systematic review found an increased perioperative and postoperative risk in patients with FVL compared to patients without FVL undergoing non-cardiac surgery, including increased thromboembolic events and graft loss and decreased graft survival (8). The same study also found that differences in the association between FVL and VTE depended on the type of surgery, where FVL increased perioperative VTE occurrence in orthopedic surgeries but not in noncancer general surgeries (8). Therefore, the current study extends the literature by addressing mortality in patients with FVL after receiving a CABG procedure. Studies that have looked at the impact of FVL on CABG, including one with 205 patients, found laboratory evidence that non-FVL patients have protection from postoperative thrombosis compared to FVL patients, but are limited in number.

Our study showed that patients with FVL who underwent a CABG procedure had hospital stays that were, on average, 1.6 days longer than those without FVL. There are several possible explanations for this. The first explanation could be that patients with FVL are more likely to have post-operative thrombotic complications that require inpatient management, thus prolonging the stay, without necessarily increasing the in-hospital mortality (8). Additionally, patients with FVL often require more complex anticoagulation management than those without FVL, and this could theoretically prolong the hospital stay for these patients (18–20).

Patients with FVL did not have significantly different total costs from their hospital stay despite on average, having longer stays than those without FVL. One possible explanation for this is nonlinear cost distribution. Several studies have found that the majority of costs from in-patient visits with surgery, including CABG procedures, is concentrated around the perioperative period and day of surgery itself and that each additional day contributes significantly less to the overall cost (22, 23). Another study investigating the cost incurred by patients who stayed more than four days in the hospital and found that length of stay has a very minimal impact on the overall cost because the majority of the cost was fixed and indirect (24). However, it is important to note that other literature revealed that patients with FVL may have increased costs with increased length of stay if they were to have another complication due to FVL, such as a venous thromboembolism (25).

Our study is not without limitations. The retrospective, observational nature of this study prevents the determination of causation. The imbalance between groups, especially in number and race/ethnicity, is a limitation that may weaken analysis and generalizability. The NIS database only analyzes in-hospital visits and does not contain any longitudinal, outpatient, or follow-up data. Since in-hospital mortality is a limited endpoint, long-term endpoints, such as 30-day mortality, would be informative in future studies. Specific thrombotic complications, such as graft thrombosis, stroke, and MI, are not included in the NIS database, but would also be worth assessing in future studies to better characterize surgical risk with FVL. The NIS database also does not identify individual patients, only hospital visits; thus, it is possible to have the same patient counted more than once. In addition, the NIS database depends on ICD-10 hospital coding. Coding errors or inconsistencies across hospitals could potentially introduce bias. This study looked at those who received a CABG procedure regardless of the number of vessels repaired, limiting our ability to see if the number of vessels repaired could have affected the outcomes; this could be an opportunity for future research. Patients with FVL also were not separated into groups based on either heterozygosity or homozygosity for the mutation, and because homozygosity is associated with a significantly higher propensity for thromboembolisms, separating these groups would be important in future analyses. The NIS database does not provide medication information for patients, so we were unable to stratify patients based on the anticoagulation regimens they received prior to and following the CABG procedures. Additionally, we are unable to account for confounding medical management, as perioperative anticoagulation likely influences post-operative outcomes in FVL patients. Finally, FVL is often not tested for, even after a DVT; thus, the FVL population could be underrepresented in this study, and the non-FVL group could have, in fact, included FVL patients, leading to potential misclassification bias (26). However, this study has considerable strengths. This study used propensity score matching to control confounding factors and reduce selection and confounding bias. Additionally, the NIS is a nationally representative database that increases the generalizability of this study.

## Conclusions

This study has established that patients with FVL who underwent a CABG procedure did not have higher rates of in-hospital mortality, despite their increased propensity for thromboses. Those with FVL did, however, have longer hospital stays but did not incur significantly higher total costs than those without FVL. This is the largest study thus far that has explored postoperative mortality in patients with FVL. These findings are important to help clinicians in making decisions related to coronary revascularization in patients with FVL and in assessing a patient's risk for a CABG procedure. These findings suggest that considering FVL status to determine an increased risk of mortality post-CABG procedure is of limited value. Future research in this area should focus on long-term post-CABG mortality in patients with FVL that expands beyond the conclusion of the hospital stay. Additionally, more research is needed to explore the reasons behind patients with FVL having longer hospital stays than those without.

## Data Availability

Publicly available datasets were analyzed in this study. This data can be found here: Data Source: Healthcare Cost and Utilization Project (HCUP)—National Inpatient Sample (NIS). Repository Name: HCUP (sponsored by the Agency for Healthcare Research and Quality, AHRQ). Direct Link to Data Access: https://www.hcup-us.ahrq.gov/nisoverview.jsp.

## References

[1] Bachar BJ Manna B. Coronary Artery Bypass Graft. Treasure Island, FL: StatPearls (2025). Available online at: http://www.ncbi.nlm.nih.gov/books/NBK507836/ (Accessed March 4, 2025). 29939613

[2] Hillis LD Smith PK Anderson JL Bittl JA Bridges CR Byrne JG 2011 ACCF/AHA guideline for coronary artery bypass graft surgery: a report of the American college of cardiology foundation/American heart association task force on practice guidelines. Circulation. (2011) 124(23):652. 10.1161/CIR.0b013e31823c074e 22064599

[3] Jawitz OK Gulack BC Brennan JM Thibault DP Wang A O'Brien SM Association of postoperative complications and outcomes following coronary artery bypass grafting. Am Heart J. (2020) 222:220–8. 10.1016/j.ahj.2020.02.002 32105988 PMC7085463

[4] Albagoush SA Koya S Chakraborty RK Schmidt AE. Factor V Leiden Mutation. Treasure Island, FL: StatPearls (2025). Available online at: http://www.ncbi.nlm.nih.gov/books/NBK534802/ (Accessed March 16, 2025). 30521223

[5] The National Human Genome Research Institute. About Factor V Leiden Thrombophilia. Bethesda, MD: National Human Genome Research Institute (2011). Available online at: https://www.genome.gov/Genetic-Disorders/Factor-V-Leiden-Thrombophilia (Accessed March 16, 2025).

[6] Moor E Silveira A Ferdinand van’t H Tornvall P Blombäck M Wiman B Coagulation factor V. Coagulation factor V (Arg506–>Gln) mutation and early saphenous vein graft occlusion after coronary artery bypass grafting. Thromb Haemost. (1998) 80(2):220–4. 10.1055/s-0037-1615176 9716141

[7] Massoudy P Thielmann M Müller-Beißenhirtz H Görlinger K Dietrich W Herget-Rosenthal S Thrombophilia in cardiac surgery-patients with symptomatic factor V Leiden. J Card Surg. (2009) 24(4):379–82. 10.1111/j.1540-8191.2008.00761.x 19040405

[8] Au E Shao I Elias Z Koivu A Zabida A Shih AW Complications of factor V Leiden in adults undergoing noncardiac surgical procedures: a systematic review. Anesth Analg. (2023) 137(3):601. 10.1213/ANE.0000000000006483 37053508

[9] Zuniga L Davis M Movahed MR Hashemzadeh M Hashemzadeh M. Association between factor-V Leiden and occurrence of acute myocardial infarction using a large NIS database. Am J Blood Res. (2023) 13(6):207–12. 10.62347/XQBZ7374 38223312 PMC10784119

[10] Mahmoodi BK Tragante V Kleber ME Holmes MV Schmidt AF McCubrey RO Association of factor V Leiden with subsequent atherothrombotic events: a GENIUS-CHD study of individual participant data. Circulation. (2020) 142(6):546–55. 10.1161/CIRCULATIONAHA.119.045526 32654539 PMC7493828

[11] HCUP-US NIS Overview. Healthcare Cost and Utilization Project (2025). Available online at: https://hcup-us.ahrq.gov/nisoverview.jsp (Accessed March 23, 2025).

[12] Jabbar AB Ismayl M Mishra A Walters RW Goldsweig AM Aronow HD Outcomes of acute myocardial infarction in patients with systemic lupus erythematosus: a propensity-matched nationwide analysis. Am J Cardiol. (2024) 233:7–10. 10.1016/j.amjcard.2024.09.014 39312991

[13] CPI Inflation CalculatorU.S. Bureau of Labor Statistics Web site (2024). Available online at: https://www.bls.gov/data/inflation_calculator.htm (Accessed March 23, 2025)

[14] Ho D Imai K King G Stuart EA. Matchit: nonparametric preprocessing for parametric causal inference. J Stat Softw. (2011) 42(8):1–28. 10.18637/jss.v042.i08

[15] Hong G. Marginal mean weighting through stratification: adjustment for selection bias in multilevel data. J Educ Behav Stat. (2010) 35(5):499–531. 10.3102/1076998609359785

[16] Harrell FE. Regression Modeling Strategies: With Applications to Linear Models, Logistic and Ordinal Regression, and Survival Analysis. Cham: Springer International Publishing (2015). Available online at: https://link.springer.com/10.1007/978-3-319-19425-7 (Accessed March 23, 2025).

[17] Stolz E Kemkes-Matthes B Pötzsch B Hahn M Kraus J Wirbartz A Screening for thrombophilic risk factors among 25 German patients with cerebral venous thrombosis. Acta Neurol Scand. (2000) 102(1):31–6. 10.1034/j.1600-0404.2000.102001031.x 10893060

[18] Douketis JD Spyropoulos AC Murad MH Arcelus JI Dager WE Dunn AS Perioperative management of antithrombotic therapy: an American college of chest physicians clinical practice guideline. Chest. (2022) 162(5):e207–43. 10.1016/j.chest.2022.07.025 35964704

[19] Sanchez JG Jiang WM Dhodapkar MM Radford ZJ Rubin LE Grauer JN. Total hip arthroplasty in patients who have factor V Leiden: elevated risks isolated to venous thromboembolism events. J Arthroplasty. (2024) 39(10):2421–6. 10.1016/j.arth.2024.05.083 38838962

[20] Zehner KM Sanchez JG Dhodapkar MM Modrak M Luo X Grauer JN. Total shoulder arthroplasty in patients with factor V Leiden. J Shoulder Elbow Surg. (2025) 34(1):18–25. 10.1016/j.jse.2024.01.041 38479723

[21] Boehm J Grammer JB Lehnert F Dietrich W Wagenpfeil S Wildhirt SM Factor V Leiden does not affect bleeding in aprotinin recipients after cardiopulmonary bypass. Anesthesiology. (2007) 106(4):681–6. 10.1097/01.anes.0000264767.41297.87 17413905

[22] Hashmi SA Raja MHR Arif A Naseem Z Pal KMB Pal KMI. Reducing post-operative length of stay, is it worth the effort? World J Surg. (2024) 48(5):1096–101. 10.1002/wjs.12115 38459712

[23] Edwards J Binongo J Mullin B Wei J Ghelani K Kumarasamy M Intensive care unit bypass for robotic-assisted single-vessel coronary artery bypass grafting. Ann Thorac Surg. (2023) 115(2):511–7. 10.1016/j.athoracsur.2022.06.044 35870521

[24] Taheri PA Butz DA Greenfield LJ. Length of stay has minimal impact on the cost of hospital admission. J Am Coll Surg. (2000) 191(2):123–30. 10.1016/s1072-7515(00)00352-5 10945354

[25] Merli G Ferrufino C Lin J Hussein M Battleman D. Hospital-based costs associated with venous thromboembolism treatment regimens. J Thromb Haemost. (2008) 6(7):1077–86. 10.1111/j.1538-7836.2008.02997.x 18445118

[26] Samim D Marques-Vidal P Alberio L Waeber G Méan M. Do hospital doctors test for thrombophilia in patients with venous thromboembolism? J Thromb Thrombolysis. (2018) 46(2):238–43. 10.1007/s11239-018-1702-6 29922879

